# Metabolic acidosis in chronic kidney disease: mere consequence or also culprit?

**DOI:** 10.1007/s00424-024-02912-5

**Published:** 2024-01-27

**Authors:** Christian Kuhn, Nilufar Mohebbi, Alexander Ritter

**Affiliations:** 1 Clinic for Nephrology and Transplantation Medicine, Cantonal Hospital St. Gallen, St. Gallen, Switzerland; 2 Praxis und Dialysezentrum Zurich-City, Zurich, Switzerland; 3 Clinic for Nephrology, University Hospital Zurich, Zurich, Switzerland

**Keywords:** CKD, Kidney transplantation, Bicarbonate, Diet, Ammonium, Citrate

## Abstract

Metabolic acidosis is a frequent complication in non-transplant chronic kidney disease (CKD) and after kidney transplantation. It occurs when net endogenous acid production exceeds net acid excretion. While nephron loss with reduced ammoniagenesis is the main cause of acid retention in non-transplant CKD patients, additional pathophysiological mechanisms are likely inflicted in kidney transplant recipients. Functional tubular damage by calcineurin inhibitors seems to play a key role causing renal tubular acidosis. Notably, experimental and clinical studies over the past decades have provided evidence that metabolic acidosis may not only be a consequence of CKD but also a driver of disease. In metabolic acidosis, activation of hormonal systems and the complement system resulting in fibrosis have been described. Further studies of changes in renal metabolism will likely contribute to a deeper understanding of the pathophysiology of metabolic acidosis in CKD. While alkali supplementation in case of reduced serum bicarbonate < 22 mmol/l has been endorsed by CKD guidelines for many years to slow renal functional decline, among other considerations, beneficial effects and thresholds for treatment have lately been under intense debate. This review article discusses this topic in light of the most recent results of trials assessing the efficacy of dietary and pharmacological interventions in CKD and kidney transplant patients.

## Introduction

Living beings depend on tight acid–base regulation as numerous vital biological processes are affected by pH. Hydrogen ion concentration may influence protein conformation, electrostatic properties, enzyme activity, protein interactions, and bioavailability of molecules. Additionally, protons are essential for the generation of adenosine triphosphate (ATP), the universal intracellular “energy provider.”

Kidneys and lungs are the main organs to guarantee acid–base homeostasis in a state of health, with the carbonic acid-bicarbonate buffer system being paramount. In this article, we follow the acid–base school of thought based on the Henderson-Hasselbalch equation, with bicarbonate being a key molecule in renal acid–base regulation, and not the Stewart`s approach in which protons are determined by strong ions [106]. Under normal conditions, the lungs eliminate roughly 15 mol of CO_2_ and the kidneys 1 mmol/kg of hydrogen ions per day, respectively [44]. These nonvolatile acids (fixed acids) are excreted by the kidneys into the urine as ammonium and titratable acids. They primarily reflect the dietary acid load and, to a lesser extent, endogenous production of organic acids. Kidneys counteract a decline in serum bicarbonate concentration via reabsorption of filtered bicarbonate, acid excretion, and regeneration of bicarbonate by ammoniagenesis [115].

Chronic kidney disease (CKD) is characterized by impaired kidney function and/or abnormal structure lasting longer than three months [129]. It is the most frequent underlying cause of metabolic acidosis (MA). MA is defined by a reduction in serum bicarbonate (often defined as < 22 mmol/l) and blood pH. In CKD, acid retention ensues when net endogenous acid production (NEAP) exceeds net acid excretion (NAE). Consequently, acid accumulates in cells and interstitial space and may eventually cause a decline in serum bicarbonate [44, 50]. The prevalence of chronic MA in CKD increases with fall in glomerular filtration rate (GFR) and is associated with increased morbidity and mortality [61].

Several studies have shown that MA is associated with CKD progression and ESKD not only in non-transplant CKD but also after kidney transplantation (KTx) [20, 61, 94, 124, 125]. ESKD comes with high morbidity and mortality. As its prevalence increases worldwide, therapeutic measures to slow renal functional decline are needed. Over the past two decades, a number of studies have been conducted to address the question of whether or not alkalinizing therapies are beneficial for kidney function. Based on the existing evidence, the general treatment recommendations focusing on the preservation of kidney function are currently the subject of controversy.

A detailed discussion of renal acid–base regulation is beyond the scope of this review article and covered elsewhere [44, 50, 115]. In addition to general aspects of MA in CKD, we will focus on the underlying mechanisms of MA in CKD and its effects on the kidney, and provide an overview of the current knowledge about the effect of alkali therapy on kidney function.

## Renal acid–base regulation

Urinary NAE is the difference between the total acid excretion (ammonium + titratable acids) and excreted bicarbonate. In the steady state, almost all of the filtered bicarbonate is reabsorbed along the nephron, with the majority (~ 70–80%) occurring the proximal tubule [44]. When urinary pH drops below 6.5, there is virtually no urinary bicarbonate excretion [89].

Kidneys eliminate the daily load of non-volatile acids primarily by urinary ammonium excretion [44, 50, 89]. Ammonium is produced in proximal tubular (PT) cells by ammoniagenesis, as systemic glutamine is metabolized to glutamate and then to α-ketoglutarate, resulting in two bicarbonate and two ammonium ions. These newly generated bicarbonate molecules are transported across the basolateral cell membranes of PT cells to reach the systemic circulation. Ammonium enters the urinary space by apical transport in place of protons via Na^+^/H^+^ exchanger 3 (NHE3) or by parallel transport of ammonia and protons via H^+^-ATPase. In the thick ascending limb of the loop of Henle, apical entry into cells occur through the Na^+^-K^+^-2Cl^−^ cotransporter (NKCC2) before ammonium reaches the interstitial space primarily via the basolateral Na^+^/H^+^ exchanger 4 (NHE4). Afterwards, it binds anionic sulfatides in the interstitial space in a reversible manner and in a concentration gradient from the inner medulla to the cortex [105]. In type A intercalated cells in the collecting duct, ammonia and protons again enter the urinary space where they form ammonium. The ammonia-specific transport proteins RhBG and RhCG (Rhesus glycoproteins) seem to play a major role in ammonia transport in the collecting duct, and not mere diffusion as previously suspected [10, 18]. Efficient urinary proton elimination is due to the high pKa of 9.2 of the ammonia buffer system, which guarantees extensive protonation of ammonia in the relatively acidic urine, ensuring continuous proton secretion [44].

Urinary titratable acids originate mainly from the systemic circulation and reach the tubular system by glomerular filtration [44]. Only a small amount derives from tubular secretion. Therefore, compensatory increase of titratable acids to raise net acid excretion is limited compared to adaptive changes in ammonium excretion [31]. Hydrogen phosphate is the most relevant titratable buffer with a pKa of 6.8, while uric acid (pKa 5.4) and creatinine (pKa 5.0) add to titratable acid excretion at more acidic urinary pH levels [89].

## Metabolic acidosis as consequence of CKD

CKD and KTx seem to share some pathogenic mechanisms of MA, while others are specific to KTx and are therefore discussed separately.

## Chronic kidney disease

Nephron loss is the hallmark of CKD progression and a consequence of interstitial fibrosis and tubular atrophy - the main histologic features of decline in renal mass (loss of functioning nephrons) for most underlying kidney diseases. As CKD progresses, glutamine uptake in the PT cells declines and the renal interstitial concentration gradient decreases, resulting in reduced ammonia secretion in the collecting duct [13, 109]. In a CKD rat model, expression of key molecules of ammoniagenesis was reduced [16]. Consequently, overall excretion of ammonia declines, but ammoniagenesis in the individual remaining nephrons increases as a compensatory mechanism [43, 64, 73, 75, 102].

Titratable acid excretion is usually maintained until CKD stage 5, with dihydrogen phosphate playing the key role, as higher serum phosphate levels cause increased glomerular phosphate filtration [73, 97]. In an analysis of the Chronic Renal Insufficiency Cohort Study (CRIC) cohort, MA and higher acid load were associated with increased phosphatemia and phosphaturia, but high NAE was not associated with augmented phosphaturic hormones [57]. This is in contrast to prior studies providing evidence that phosphaturic hormones may play a relevant role in phosphorus homeostasis in MA. In vitro, it was demonstrated that MA directly increases fibroblast growth factor 23 (FGF23) mRNA and protein expression in mouse bone [62]. Parathyroid hormone (PTH) levels were reported to be augmented in cell cultures (HEK-293 cells and bovine parathyroid cells) as a result of altered pH-dependent responsiveness of the calcium-sensing receptor [17]. However, a rat MA model by ammonium-chloride ingestion demonstrated that chronic MA resulted in increased phosphaturia and reduced proximal tubular sodium-phosphate cotransporter activity, which was independent of PTH activity in chronic MA but shown to be attenuated by dietary phosphate intake [4].

Finally, hemoglobin like bicarbonate also serves as a buffer system in the blood, but is decreased in renal anemia because of impaired erythropoietin production in advanced CKD, which therefore may potentially contribute to the occurrence of MA.

As GFR declines, the prevalence of MA increases, rising from 7% in CKD stage 2 to 37% in CKD stage 4 in the CRIC study [90]. In CKD, a normal or increased (in advanced CKD) serum anion gap with normal serum chloride is typically found [128]. The increased anion gap is the result of the accumulation of titratable acids with declining GFR.

### Subclinical metabolic acidosis

Current literature describes the concept of a so called “eubicarbonatemic metabolic acidosis,” a subclinical metabolic acidosis that appears in earlier CKD stages, preceding overt MA with slightly reduced serum bicarbonate but levels still in the normal range [3, 88, 123]. This concept assumes a continuum of MA development with various compensatory mechanisms attempting to maintain acid–base balance, rather than a clear threshold above which acid accumulates as indicated by the arbitrary definition of “normal” serum bicarbonate levels. In CKD, kidneys fail to keep up with the daily load of fixed acids deriving from metabolism. The acids originate in cells and have to pass the interstitial space and several layers of buffer and lead to a decline in serum bicarbonate in the blood, the fluid where we typically measure acid–base parameters. To capture an early state of subclinical acid accumulation, urinary measurements other than a profound decline in serum bicarbonate may be more sensitive, such as urinary ammonium excretion. It could be argued that these subclinical changes affecting blood acid–base parameters to a lesser extent are therefore unlikely to be relevant for CKD progression or other clinical outcomes. However, several studies point into the direction that these early stages of acid retention may have clinical implications. In the NephroTest cohort, CKD stage 1–4 patients with low ammonium excretion had a higher risk for ESKD even without overt MA [111]. In the African American Study of Kidney Disease and Hypertension (AASK), a low urinary ammonium excretion in a state of normal serum bicarbonate indicating acid retention was associated with an increased risk for end-stage kidney disease (ESKD), death, and overt acidosis at 1 year compared to those with normal serum bicarbonate and higher ammonium excretion [91]. The pH-sensitive metabolite citrate is another interesting candidate marker to identify early acid retention in subclinical MA [41]. The citrate cycle serves the oxidative degradation of organic substances for the purpose of energy production and the provision of intermediates for biosynthesis. Citrate metabolization leads to a net gain in bicarbonate. Citrate excretion declines in MA [54]. It has been demonstrated that urinary citrate-to-creatinine ratio is sensitive to changes in GFR, acid stress, and alkali therapy and that it might be superior to serum bicarbonate to detect acid accumulation [35]. An association of acid retention and the effect of reduction in acid load by fruit and vegetable diet on 8 h urinary citrate excretion was demonstrated in CKD stage 1 and 2 patients [40]. However, it has to be taken into account that citrate serves various physiologic functions and is not specific to acid–base regulation. Furthermore, the relevance of hypocitraturia on clinical CKD outcomes remains to be examined. To date, the ideal metabolic parameter or panel of metabolites to capture an early state of acid retention have not been identified yet.

NEAP is a result of dietary intake of fixed acids (mainly animal protein) and alkali precursors (organic anions like citrate and acetate). The Western diet is rich in animal protein, including the sulfur-containing amino acids methionine and cysteine, which are metabolized to sulfuric acid and therefore represent an “acid stress”. In CKD, dietary acid load is a key factor that may lead to either subclinical or overt MA.

A better understanding of acid–base regulation in the kidney is needed to clarify whether subclinical acid accumulation in renal tissue is as relevant a factor as is often discussed.

## Kidney transplantation

**Fig. 1:**
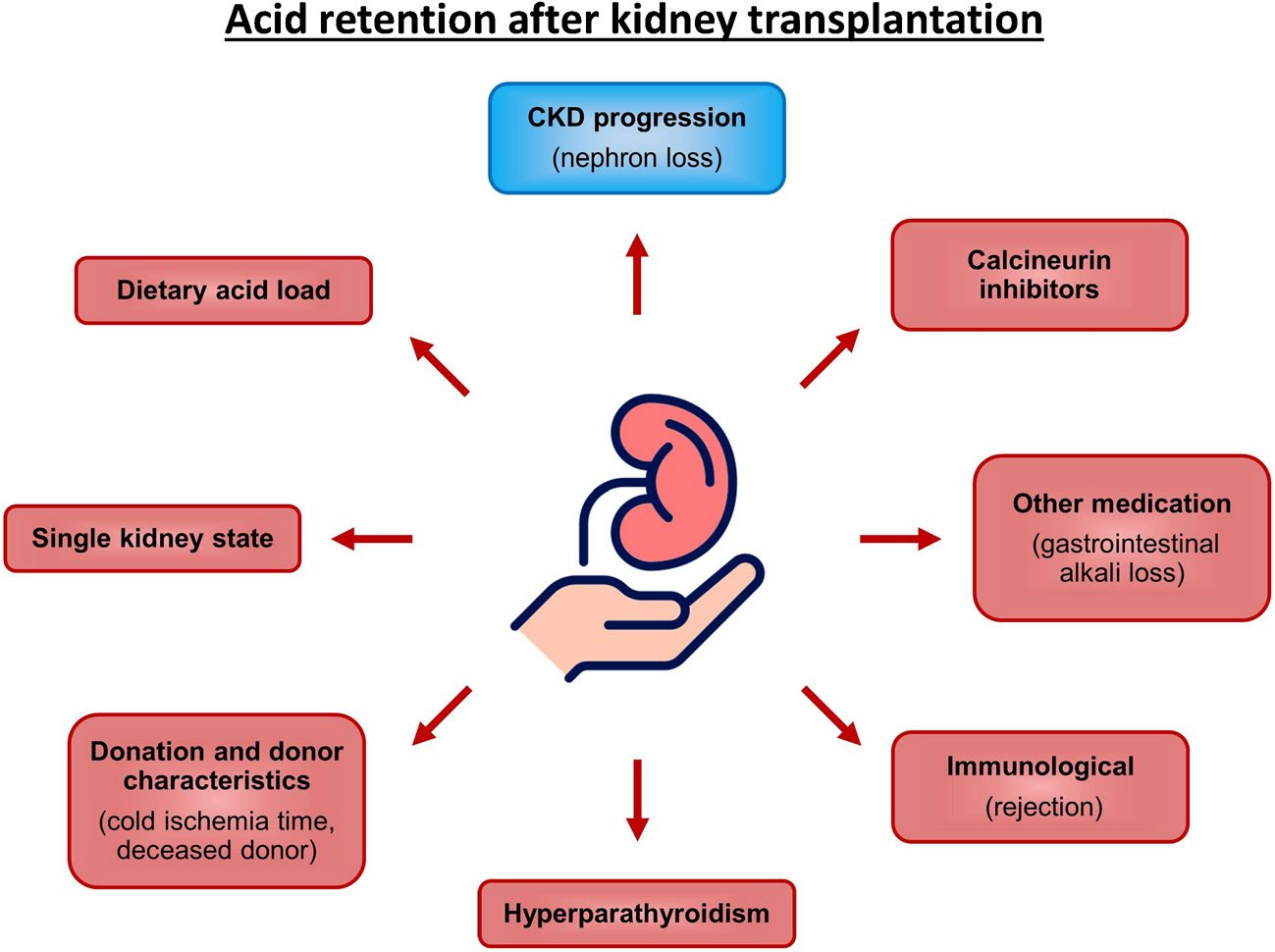
Additional factors likely contributing to metabolic acidosis after kidney transplantation. Apart from CKD progression with nephron loss (blue), some factors are specific to kidney transplantation, and contribute to acid retention (red). Calcineurin inhibitors impair tubular adaptation capacity and tubular acid handling, while medication such as mycophenolate and antibiotics may cause fecal alkali loss due to diarrhea. Hyperparathyroidism can lead to proximal RTA type II early after kidney transplantation and usually resolves. Donor and donation characteristics (deceased donor transplantation, cold ischemia time) may be associated with metabolic acidosis; rejection is controversial. The single kidney state with alterations in chemical and electrical gradients may impair net acid excretion. Dietary acid load may unmask incomplete renal tubular acidosis

Among KTx patients, the prevalence of MA is high, ranging from 12 to 58%, and it is seen at higher GFR levels compared to non-transplant CKD patients [68]. This is likely due to additional pathophysiologic mechanisms after organ transplantation as depicted in Fig. 1. The typical presentation of MA in KTx also differs. It is commonly manifesting as renal tubular acidosis (RTA) with normal anion-gap and normal or high serum chloride levels [98]. Distal (classic) RTA type I and distal (hyperkalemic) RTA type IV are the predominant forms. However, proximal RTA type II may be found early after KTx as a result of tubular damage and hyperparathyroidism. It usually resolves within the first months after KTx.

The use of calcineurin inhibitors (CNIs) as a mainstay of most immunosuppressive regimens seems to be a major cause of RTA occurrence after KTx. CNI treatment was associated with chronic MA in a number of studies [12, 45, 81, 98, 126]. This seems to happen in a dose dependent manner [59, 103]. Tubular function can be impaired by cyclosporine A and tacrolimus as was demonstrated in humans and animals [45, 70, 116]. In animal experiments, changes in electrolyte transport resulting in RTA were identified. Cyclosporine A seems to inhibit the peptidyl-prolyl cis–trans isomerase activity of cyclophilin in distal tubular type B intercalated cells and thereby reduces their cellular adaptation capacity [116]. Tacrolimus seems to affect the expression and distribution of key transport proteins like the anion exchanger AE1, sodium bicarbonate cotransporter NBCe1, and the vacuolar proton pump [70]. Furthermore, tacrolimus causes hypertension, hyperkalemia, and acidosis by activating the sodium chloride cotransporter (NCC) and its regulatory kinases [48].

After KTx, additional factors may contribute to MA. Non-anion gap MA can be provoked by drugs, in particular mycophenolate and antibiotics, that often cause diarrhea with ensuing fecal alkali loss. Whether immunological factors like rejection play a role is controversial as results are conflicting [94]. Hyperchloremic MA was suspected to be an early sign indicating rejection [7, 72]. For example, in Sjogren`s syndrome, autoantibodies can cross-react with intercalated cells and potentially cause RTA [29]. A similar immunologic mechanism was suspected for antibody mediated rejection episodes. However, in KTx, there is no clear association of acute rejection and RTA throughout the existing literature [12, 81, 98]. Reduced immunoactivity of the vacuolar proton pump (and AE1), initially attributed to rejection in a patient with MA, was also detected in another study independent of acute rejection [21, 86]. Donation and donor characteristics could potentially be relevant but evidence is rather limited so far [94]. In a few studies, MA was more frequent in patients with organs from deceased donors. Longer cold-ischemia time, the time from organ retrieval to reperfusion, was associated with MA three months post KTx.

Alterations in chemical and electrical transtubular gradients, caused by increased perfusion of the transplanted kidney due to the single kidney state, could be an additional mechanism causing a decrease in NAE, as was shown for patients with nephrectomy [19].

As shown in a large cohort, dietary acid load with an increase in NAE is a relevant factor in MA occurrence after KTx [112]. It could also unmask incomplete RTA, a tubular defect that becomes only overt after acid stress.

## Impact of metabolic acidosis in chronic kidney disease

MA in CKD is associated with several organ dysfunctions, which may take time to fully develop (3). CKD appears to influence the cellular response to MA. In particular, muscle wasting and bone disease as consequence of acid retention are relatively well studied. Furthermore, hypoalbuminemia, inflammation, impaired glucose tolerance, and other hormonal changes (for example growth hormone, insulin-like growth factor 1, thyroid hormone) have been described. Mortality has been shown to be increased in CKD patients with MA. Furthermore, an association of MA in CKD with cognitive dysfunction is discussed [51]. In KTx, a few mainly observational studies identified associations with impaired bone metabolism, cardiovascular events, and mortality [94]. The increasing prevalence with declining kidney function - both in the non-transplant CKD as in the KTx setting - clearly indicates that MA occurrence is a consequence of declining kidney function. However, to assess the question whether MA is only a marker of impaired kidney function or indeed also a “culprit” that promotes disease progression by nature observational studies are insufficient. MA could simply be an indicator of disease severity. Answering the question of causality requires a deeper understanding of pathophysiological effects of MA on renal tissue and interventional studies that demonstrate either a negative impact of MA on kidney functional decline or a positive effect by MA correction through alkali therapy. In the following section, we aim to provide an overview of the current state of knowledge about this question.

## Effects of metabolic acidosis on the kidney

**Fig. 2:**
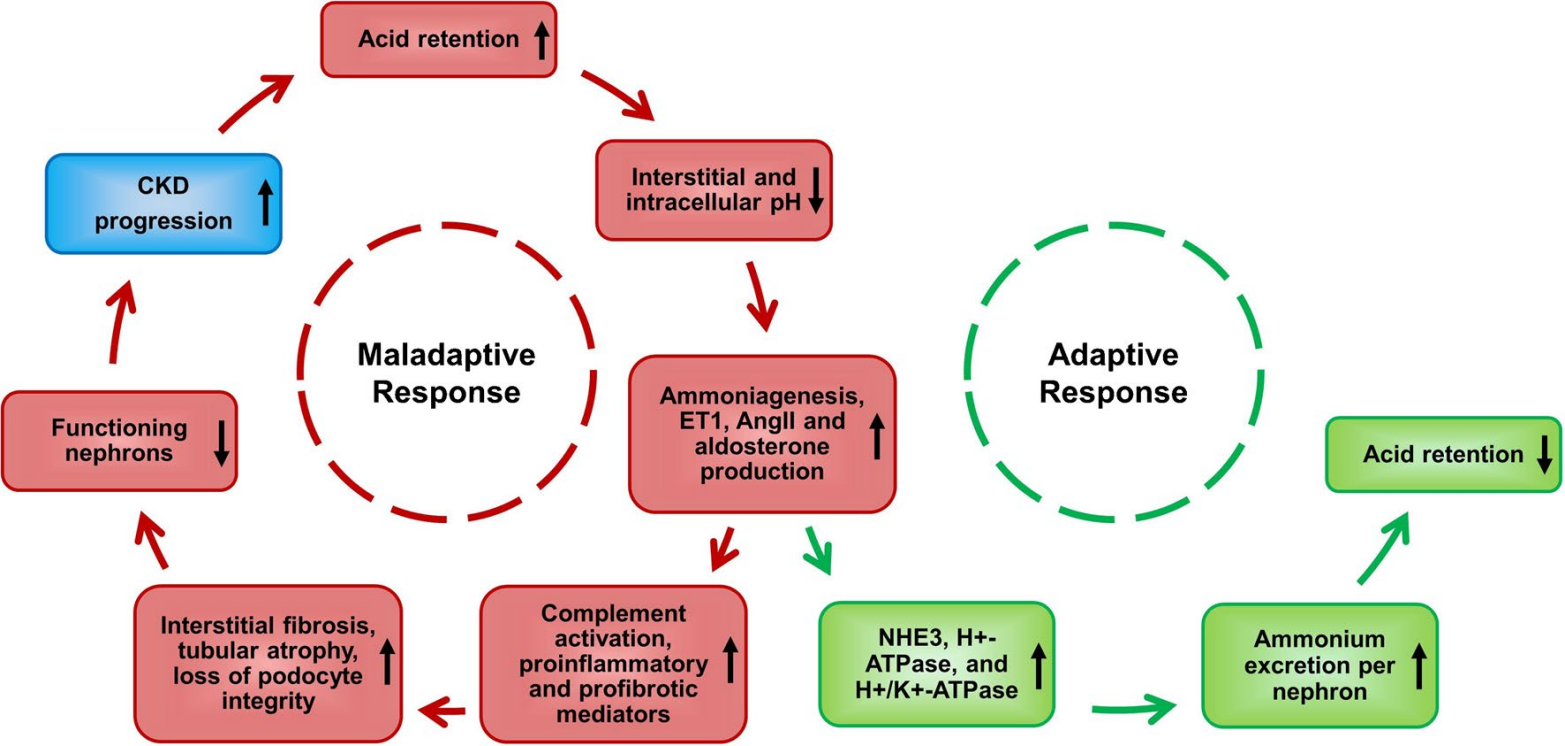
Consequences of metabolic acidosis on the kidney and adaptive responses. Vicious cycle initiated by acid retention leading to a cascade of events contributing to progression of chronic kidney disease (CKD). Metabolic acidosis (MA) triggers increased ammoniagenesis in remaining nephrons, endothelin-1 (ET1) production, and activation of the renin-angiotensin system (RAS), subsequently promoting complement activation by ammonium, as well as generation of proinflammatory and profibrotic mediators. Interstitial fibrosis and tubular atrophy, and loss of podocyte integrity, culminating in loss of functioning nephrons is a result. This exacerbates acid retention, perpetuating the maladaptive response. In the adaptive response, the kidney upregulates various acid transporters, collectively leading to increased ammonium excretion and hence decreased acid retention. Figure modified after [123]

The physiological adjustments mentioned earlier enhance the removal of acid from the body, thereby assisting in the restoration of serum bicarbonate levels. However, these same processes could potentially accelerate the development of chronic kidney disease (CKD) as shown in Fig. 2. Chronic MA upregulates various intrarenal paracrine hormone systems, including angiotensin II (AngII), aldosterone, and endothelin-1 (ET1). Collectively, they augment NAE, but may come at the expense of activation of deleterious downstream pathways.

The renin-angiotensin system (RAS) is both systemic and intrinsic to the kidney, the latter operating independently of the systemic RAS. PT cells produce angiotensinogen, which is subsequently secreted into the filtrate, where it is activated to AngII [78]. Within the PT, AngII augments ammoniagenesis, which is further potentiated in the distal tubule by enhanced Na + /H + exchange, H + /K + -ATPase, and H + -ATPase activity, leading to increased NAE [122]. Furthermore, the basolateral Na^+^/HCO_3_-cotransporter (NBC1) is upregulated by AngII, enhancing bicarbonate reabsorption in the PT [110]. Notwithstanding its short-term benefits, there is growing evidence that sustained intrarenal AngII activity may mediate interstitial inflammation, subsequently culminating in fibrosis and tubular atrophy [95, 100]. It has also been shown that AngII promotes podocyte apoptosis by downregulation of the synthesis of negatively charged proteoglycans and nephrin [96]. Intriguingly, treatment with systemic ACE-inhibition has no effect on the kidney-inherent RAS [78, 79]. Whether bicarbonate loading by dietary interventions or oral alkali supplementation are able to attenuate intrarenal AngII levels, as assessed by measuring urinary angiotensinogen as a surrogate, remains debated [11, 39]. Given that urinary angiotensinogen mirrors albumin excretion, and thereby might simply reflect glomerular filtration and renal handling of both proteins, urinary renin has been proposed as a more accurate indicator of intrarenal RAS [113]. The synthesis of Aldosterone, another part of the RAS, is also augmented in states of metabolic acidosis, even after systemic blockade of AngII synthesis [47]. Aldosterone not only promotes distal-tubular acidification, but also exhibits hemodynamic and profibrotic properties, including reactive oxygen species, cumulatively undermining kidney integrity [55, 85]. Studies that investigated if bicarbonate supplementation reduces both plasma and urine aldosterone excretion have been promising [121]. However, the protective effects on the kidneys involve more than just aldosterone, as blocking aldosterone alone has a lesser effect on GFR compared to alkali supplementation [119]. Taken together, the upregulation of the RAS is heavily inflicted in profibrotic pathways leading to CKD, which may be alleviated by alkali supplementation, but the reflection of urinary markers as index of intrarenal RAS remains controversial.

Another hormone that seems to be inflicted in MA-mediated kidney fibrosis is ET1, a peptide predominantly produced by the endothelium, though glomerular epithelial and mesangial cells also contribute to synthesis [25, 32, 120]. Beyond its well-known function as potent vasoconstrictor, ET1 underpins several renal functions to maintain homeostasis, including maintaining podocyte integrity and increasing proximal and distal tubular acidification in response to a dietary acid challenge [28, 118]. Analogous to AngII, while ET1’s short-term roles enhance acid excretion, prolonged elevations may have deleterious consequences. In vitro studies have demonstrated that ET1 promotes the synthesis of fibronectin and collagen 5. Additionally, sustained ET1 levels are linked with inflammation, fibrosis, and podocyte effacement [83, 93]. Furthermore, ET1 increases the production of AngII, which reciprocally stimulates renal ET1 formation, creating a deleterious positive feedback loop [6, 56]. Blocking of the endothelin A receptor by a selective antagonist was shown to reduce the risk of renal events in patients with diabetes and CKD in a double-blind, randomized, placebo-controlled trial [46].

In summary, the hormonal renal response to MA, while ensuring acid–base homeostasis, paradoxically becomes maladaptive in chronic MA and may induce deleterious inflammatory and profibrotic effects. Accumulating data advocate that in MA, alkali substitution, whether dietary or pharmacological, might attenuate these adverse effects.

Ammonium-mediated activation of the complement cascade seems to further contribute to CKD progression. As indicated above, overall ammonia synthesis decreases along with nephron loss as CKD progresses, whereas single-nephron ammoniagenesis of the remaining nephrons increases to compensate for the loss [58]. Ammonia reacts with the complement C3 to form a convertase for the alternative complement pathway, which has been postulated to be an independent factor for inflammation. In a remnant kidney rat model, bicarbonate supplementation led to less impairment of tubular function (as assessed by proteinuria), reduced histological signs of tubulo-interstitial injury, and diminished deposition of complement C3 and the membrane attack complex (C5b-9) [74]. This suggests that the compensatory increase in single-nephron ammoniagenesis causing complement-mediated inflammation could further promote decline in kidney function in CKD. However, caveats remain about the infliction of complement activation in MA, since complement activity seems not to correlate with the increase in ammoniagenesis, and NaCl used in the control group might be more prohypertensive than NaHCO_3_ used for correction of acidosis, as discussed by Mannon and O`Connor [66].

MA is also closely linked to glucose metabolism. Alongside the liver, the kidneys have the capacity to synthesize glucose and play a pivotal role in regulation blood glucose levels. After an overnight fast, the kidneys are responsible for ~ 40% of the body’s gluconeogenesis, a figure that can increase to as much as 50% after prolonged periods of starvation [34, 80]. In the presence of acidosis, there is a shift away from using substrates of gluconeogenesis such as lactate (which is typically the primary substrate) towards the utilization of glutamine [69, 84]. In both healthy humans and animal research models, the activation of ammoniagenesis and gluconeogenesis in response to acidosis leads to an elevation in renal glucose production [1, 104]. The α-ketoglutarate produced through the renal breakdown of glutamine is primarily converted into glucose, a process that involves the enzyme phosphoenolpyruvate carboxykinase. This enzyme becomes upregulated during acidosis and increases the ability of the kidneys to remove lactate, whereas extrarenal lactate clearance decreases with acidosis [2, 15, 63]. Consequently, in conjunction with ammoniagenesis, renal gluconeogenesis results in both glucose production and the excretion of acid, thereby contributing significantly to the maintenance of systemic acid–base balance [23, 24]. It’s worth noting that while this mechanism works in individuals with preserved kidney function, in patients suffering from CKD, the kidneys extract only reduced amounts of glutamine, even after an oral glutamine loading [108, 117]. This suggests that in CKD, glutamine delivery may not be a rate-limiting step for ammoniagenesis. More probably, the downregulation of gluconeogenesis enzymes acts as the culprit. This has recently been corroborated in a study using transcriptomics in kidney biopsies of CKD patients as well as in a CKD mouse model, where the downregulation of gluconeogenesis enzyme genes was still observed when measured at the cellular level, and therefore independent of reduction in tubular mass [114].

Acidosis has broader implications on the metabolic functions of the kidneys than just gluconeogenesis. Mitochondria, for instance, play a pivotal role in generating adenosine triphosphate (ATP) through oxidative phosphorylation. They are particularly abundant in PT cells and the thick ascending limb due to the high energy demand for solute reabsorption. Additionally, the tricarboxylic acid cycle (TAC, also known as the citric acid cycle) takes place within mitochondria. This cycle oxidizes acetyl-CoA derived from carbohydrates, fats, and proteins, reducing NAD^+^ to NADH, which then participates in oxidative phosphorylation. Studies indicate that when compared to healthy controls, TAC activity is significantly reduced in CKD patients, which supports the perspective that CKD may be seen as a state of mitochondrial dysfunction [42, 101]. One small study employing metabolomics found that alkali treatment seems to restore circulating TAC intermediates, indicating a potential protective mechanism against CKD progression [99]. Another recent investigation employed transcriptomics on kidney transplant recipients, both with and without acidosis, revealing significant alterations in 40 transcripts mainly associated with PT amino acid and lipid metabolism. Notably, alkali treatment successfully reversed three of these altered transcripts [52]. A separate study involving mice subjected to a sudden acid load identified an immediate shift in the nicotinamide adenine dinucleotide (NAD) redox balance within the mitochondria of PT cells. This led to lipid build-up in PT cells, subsequently causing acute tubular damage. Yet, increasing blood pH with intravenous bicarbonate greatly enhanced tubular functionality [14]. Upregulation in genes encoding several proinflammatory cytokine proteins and metallopeptidases has been found in another study using Madin-Darby canine kidney cells subjected to an acidic milieu [87]. Lastly, a recent study delved into new mechanisms that might explain the advantage of alkali treatment in both an oxalate-induced murine crystallopathy model and human biopsies of kidney transplant recipients [82]. Bicarbonate supplementation reestablished multiple deranged cell metabolism pathways involved in lipid, cholesterol, and iron homeostasis. Furthermore, it led to the reestablishment of α-Klotho levels, which may limit the expression of adhesion molecules in injured kidneys and thereby downregulate leukocyte recruitment.

However, despite the above findings suggesting that MA is indeed an independent culprit in the progression of CKD, there are studies that call this result into question. For example, one study subjected rats to a dietary hydrochloric acid load for 14 weeks [107]. Although there was an expected decrease in urine pH as a surrogate of upregulated ammoniagenesis, GFR was identical to the control group, and kidney histology assessed by light microscopy was normal. The same research group performed 5/6 nephrectomies in rats, and although one group received oral bicarbonate to correct acidosis, there was no difference compared to the control group in terms of GFR or histological findings. In another study, chronic MA even protected against CKD progression in phosphate-loaded 5/6 nephrectomized rats [53]. Finally, the hypothesis of a direct profibrotic effect of hormone upregulation on the deterioration of renal function has been challenged and it has been suggested that hemodynamic pathways may dominate, possibly ending in a common downstream pathway of damage such as fibrosis or inflammation. For example, upregulation of AngII and aldosterone in the setting of salt-restriction does not appear to lead to kidney injury [22]. In addition, in a two-kidney, one-clip hypertensive model, inflammatory cells, and histological signs of CKD after 11 weeks were observed almost exclusively in the unclipped kidney exposed to hypertension, again suggesting that AngII without concomitant hypertension is not a main culprit leading to kidney injury.

Taken together, the relationship between MA and CKD progression is complex and multifaceted. While there is substantial evidence supporting MA’s role in exacerbating CKD through hormonal imbalances and inflammatory pathways, this perspective is not definitive, with some studies suggesting that factors like hemodynamic changes might be more influential in kidney injury, questioning the direct culpability of MA.

## Correction of metabolic acidosis in CKD

The key question from a kidney point of view is whether a correction of MA, either through dietary interventions or pharmacological therapies, will help slow down the decline in renal function. What treatment recommendations should be given in the non-transplant CKD- or the KTx-setting? To address these questions, a number of trials have been undertaken in the last two decades.

## Chronic kidney disease

### Dietary interventions

Several observational studies have shown a beneficial effect of lower dietary acid load on kidney function. For example, a study in the National Health and Nutrition Estimation Survey (NHANES) III population detected a threefold increased risk of ESKD in CKD participants in the highest tertile of dietary acid load [5]. In the last decade, there have been a few dietary intervention trials assessing the effect of fruits and vegetables (F/V) on CKD progression across different CKD stages and baseline serum bicarbonate levels. In early stages of hypertensive nephropathy, the group of Wesson could demonstrate a beneficial effect of reduced dietary acid load from a diet rich in F/V despite normal serum bicarbonate in CKD stage 2 (but not in CKD stage 1) [37]. In a short controlled trial of 30-day duration, they examined 199 CKD stage 1 and 2 patients with plasma total CO_2_ ≥ 24.5 mmol/l and achieved 50% reduction in potential renal acid load (PRAL) through F/V consumption. There was no change in serum bicarbonate levels, but a decrease in urinary albumin-to-creatinine ratio (ACR), transforming growth factor-β1 (TGF-β1) and N-acetyl beta-D-glucosaminidase (NAG) in CKD stage 2 patients treated with F/V or sodium bicarbonate. In a trial with 108 CKD stage 3 patients and plasma total CO_2_ of 22–24 mmol/l, an intervention with F/V or sodium bicarbonate over 3 years preserved eGFR and reduced ACR [39]. In 71 CKD stage 4 patients with plasma total CO2 levels < 22 mmol/l at baseline, ACR, NAG, and TGF-β1 were lowered under F/V or sodium bicarbonate treatment over 1 year [38]. Weight and blood pressure also decreased in the F/V treatment arm, while there was no effect on eGFR. A recent meta-analysis comprising six trials investigating dietary interventions (one with additional oral alkali supplementation) compared to controls found low- to moderate-certainty evidence that reduced dietary acid intake slows renal functional decline in CKD with MA [76]. Plant-based protein is preferred over animal protein because of sulfur-containing amino acids of the latter. However, in progressing CKD, the risk of hyperkalemia has to be taken into account. F/V diet may help preserve kidney function not only by mitigating MA. It has to be considered that there might be other dietary reasons apart from the reduced dietary acid load and that the beneficial effect could at least to some extent be falsely attributed to it. Other mechanisms like lowering blood pressure and body weight or lower salt and phosphate content could also play a role in beneficial renal outcomes. High protein intake has been shown to cause kidney hypertrophy and augment glomerular pressure and hyperfiltration with negative impact on kidney function and proteinuria [33, 60]. Since high protein intake and high NAE go hand in hand, it is difficult to analyze the different contribution of proteins/amino acids and acid load on kidney function. However, due to adverse outcomes, a very strict protein restriction is not recommended in CKD.

### Pharmacological therapies

In recent years, a number of randomized controlled trials of varying size and study duration were undertaken in the non-transplant CKD population of different CKD stages and serum bicarbonate levels investigating the effect of oral alkali therapy on kidney function. In most cases, the treatment consisted of sodium bicarbonate. A recent systematic meta-analysis comprised 15 sodium bicarbonate trials (total of 2245 participants, median follow-up of 1 year) - some with placebo control and others with open-label design [49]. It showed low certainty evidence that sodium bicarbonate slows renal functional decline and reduces the risk of ESKD. Results were in line with an earlier systematic meta-analysis that also comprised a trial with sodium citrate treatment and found low-to-moderate certainty evidence for these outcomes [76].

Sample size of the majority of these trials was rather small and study designs not always rigorous. The largest trial of the latest meta-analysis originating from Italy (UBI Study), that was also given the highest weight, comprised 795 CKD stage 3–5 patients in multiple centers with 3 years of follow-up [30]. Results demonstrated a significant difference in the composite primary outcome (doubling of serum creatinine, time to renal replacement therapy, or all-cause mortality) between the sodium bicarbonate and standard care group in favor of alkali therapy. We consider the open label design and the lack of standardization of care among centers to be the main limitations that might have caused treatment bias.

Another earlier open label trial in 134 patients with CKD and MA from the UK demonstrated a slower decline in creatinine clearance, less patients with rapid decline in creatinine clearance (> 3 ml/min/year), and less patients that experienced ESKD in patients randomized to sodium bicarbonate treatment compared to standard care [26]. Apart from the rather small sample size, the single-center design and again the lack of placebo control may be viewed as main limitations.

In a small single-center randomized-controlled trial with hypertensive nephropathy patients in CKD stage 2 with albuminuria (> 200 to < 2000 mg/g Crea) over 5 years with 40 patients per treatment arm, patients in the sodium bicarbonate group experienced a slower cystatin C-based eGFR decline compared to placebo or sodium chloride treated patients [65]. Interestingly, on average, these patients did not have overt MA.

Another trial from the UK (BiCARB), which was conducted in 27 nephrology and geriatric medicine departments focused on a geriatric population (≥ 60 years) and involved 300 CKD patients with mild MA, was primarily designed to assess the impact of bicarbonate on physical function. Compared to placebo, the study did neither find improvement in physical function nor in renal function (assessed as secondary outcome) at 1 year [9]. Patients were followed up to 2 years, and there were more adverse events in the bicarbonate group. In our view, a major limitation of the study is the pragmatic study design, leading to cross-over between treatment groups and thereby limiting the ability to answer the true effect of bicarbonate on these outcomes. Over the study period, serum bicarbonate levels leveled off between treatment groups. This makes it almost impossible to work out the true physiological effect of bicarbonate administration, even though the pragmatic study design may correspond to the treatment reality of many “real world patients.”

Sodium loading and consequent increase in volume retention and blood pressure is always a concern with this treatment. However, a recent meta-analysis of 2110 patients in 14 trials did not identify evidence with moderate certainty that sodium bicarbonate negatively affects blood pressure [8].

According to preliminary results, a phase 3 trial of the novel agent veverimer (VALOR-CKD) to bind hydrochloric acid in the gastrointestinal tract in 1480 CKD patients with MA (eGFR 20–40 ml/min/1.73 m^2^) did not show evidence of benefit on a composite renal endpoint (ESKD, eGFR decline ≥ 40%, death due to kidney failure) compared to placebo [27, 67]. However, the trial seems to have failed to discriminate serum bicarbonate levels as anticipated between treatment groups.

To date, robust data from large, well-designed studies with sufficient follow-up that manage to maintain a difference in serum bicarbonate over the entire study duration are lacking to give us a final answer for a general treatment recommendation.

Based on these uncertainties, the preliminary novel “KDIGO 2023 Clinical Practice Guideline for the Evaluation and Management of Chronic Kidney Disease for public review” intends to lower the serum bicarbonate threshold for treatment from < 22 to < 16 mmol/l [119]. It particularly stresses the results of the so far largest randomized controlled bicarbonate trial in CKD (BiCARB), which in our opinion is problematic due to the methodological limitations described above [9].

In this respect, the so-called BASE pilot trial investigating safety, tolerability, and adherence as well as pharmacodynamics of two different dosages of oral bicarbonate was an important initiative to establish a solid basis for future large scale randomized controlled trials with capability to detect moderate-sized treatment effects [77, 92]. Although the pendulum may currently be swinging towards lower bicarbonate levels as trigger for intervention, it appears that the final word regarding alkali therapy in CKD with MA does not seem to have been spoken.

## Kidney transplantation

### Dietary interventions

After kidney transplantation, in a Dutch cohort of 642 kidney transplant recipients (KTRs) with a median follow-up of 5.3 years, an association of dietary acid load was found with a higher risk of doubling of plasma creatinine or graft failure - NEAP measured with food frequency questionnaires and urinary excretion [127]. In another cohort study, a Mediterranean diet was associated with better allograft outcomes [36]. To our knowledge, there are no interventional trials regarding dietetic interventions on MA on allograft outcomes in KTRs so far.

### Pharmacological therapies

In kidney transplantation, an increasing number of observational studies have seen an association of MA with decreasing allograft function or allograft failure [12, 81, 94]. However, to date, there is only one randomized-controlled trial in the kidney transplant setting (Preserve-Transplant Study) [71]. Surprisingly, this multicentric placebo-controlled trial over 2 years did not find a beneficial effect of sodium bicarbonate on eGFR slope as surrogate renal endpoint in 240 KTRs with MA underlining the importance of randomized evidence before treatment recommendations are made. Study participants with an eGFR of 15–89 ml/min/1.73 m^2^ at least 1 year after KTx were included. A significant difference in serum bicarbonate levels was maintained throughout the study duration between treatment groups. Safety concerns, including increases in blood pressure due to sodium load, were not identified. Currently, recruitment for another smaller, placebo-controlled trial in 120 KTRs investigating the effect of sodium bicarbonate therapy over 1 year duration on surrogate markers of graft function (and cardiovascular disease) is ongoing and will contribute further evidence in the KTx-setting (ClinicalTrials.gov ID NCT05005793).

Why trial results on kidney function differed between the transplant and many trials in the non-transplant CKD population could have several reasons. A main aspect could be that it is more difficult to overcome the additional pathophysiologic antagonistic mechanisms (probably mainly caused by CNIs among others) to reduce acid retention not only in blood but also down to the interstitial and cellular space of the transplanted kidney. Whether additional measures to sodium bicarbonate treatment like for example dietetic interventions would help to further reduce acid retention and to slow functional graft decline remains to be determined. At the moment, based on the existing evidence in KTx, a general recommendation for sodium bicarbonate treatment of MA to preserve allograft function cannot be given. Official treatment guidelines are lacking for KTRs with MA so far.

## Conclusion

Metabolic acidosis is a frequent health issue in non-transplant CKD and KTx. The underlying pathophysiology is only partially overlapping. In our opinion, the evidence of MA being more than just a consequence of CKD but also a culprit cannot be neglected. However, treatment effects of alkali therapy in non-transplant CKD seem to be smaller than hoped for and smaller than suggested in early clinical studies. To date, there is low(-to-moderate)  certainty evidence that oral alkali therapy or dietary interventions are beneficial for renal function. Treatment recommendations are not straightforward and clear cut-offs, when to begin treatment and what treatment targets are to be achieved are not well defined and currently under vivid debate. Further evidence from robust trials is therefore needed. In this discussion about treatment of this fragile patient population, the multifaceted role of acid–base homeostasis on diverse bodily functions apart from the kidney should not be forgotten. A more in depth understanding of metabolic changes originating from or being a cause of MA may help in the future to detect earlier stages of acid retention, to find new potential therapeutic targets and to identify patients at risk that might benefit from therapy. Based on current knowledge, for KTRs, sodium bicarbonate treatment cannot be generally recommended and randomized evidence for dietary advice is missing. Hopefully, in the coming years, research will provide us with answers to these open questions.

## Data Availability

No datasets were generated or analysed during the current study.
